# Predicting renal disease progression in a large contemporary cohort with type 1 diabetes mellitus

**DOI:** 10.1007/s00125-019-05052-z

**Published:** 2019-12-05

**Authors:** Marco Colombo, Stuart J. McGurnaghan, Samira Bell, Finlay MacKenzie, Alan W. Patrick, John R. Petrie, John A. McKnight, Sandra MacRury, Jamie Traynor, Wendy Metcalfe, Paul M. McKeigue, Helen M. Colhoun, on behalf of the Scottish Diabetes Research Network (SDRN) Type 1 Bioresource Investigators and the Scottish Renal Registry

**Affiliations:** 1 Usher Institute of Population Health Sciences and Informatics, University of Edinburgh, Edinburgh, UK; 2 MRC Institute of Genetic and Molecular Medicine, The University of Edinburgh, Western General Hospital, Crewe Road South, Edinburgh, EH4 2XU UK; 3 Renal Unit, Ninewells Hospital, Dundee, UK; 4 Birmingham Quality/UK NEQAS, University Hospitals NHS Foundation Trust, Birmingham, UK; 5 Royal Infirmary of Edinburgh, NHS Lothian, Edinburgh, UK; 6 Institute of Cardiovascular and Medical Sciences, University of Glasgow, Glasgow, UK; 7 Western General Hospital, NHS Lothian, Edinburgh, UK; 8 Department of Diabetes and Cardiovascular Science, University of Highlands and Islands, Inverness, UK; 9 NHS Greater Glasgow and Clyde, Glasgow Renal and Transplant Unit, Queen Elizabeth University Hospital, Glasgow, UK; 10 NHS Fife, Kirkcaldy, UK

**Keywords:** Clinical science, Epidemiology, Nephropathy

## Abstract

**Aims/hypothesis:**

The aim of this study was to provide data from a contemporary population-representative cohort on rates and predictors of renal decline in type 1 diabetes.

**Methods:**

We used data from a cohort of 5777 people with type 1 diabetes aged 16 and older, diagnosed before the age of 50, and representative of the adult population with type 1 diabetes in Scotland (Scottish Diabetes Research Network Type 1 Bioresource; SDRNT1BIO). We measured serum creatinine and urinary albumin/creatinine ratio (ACR) at recruitment and linked the data to the national electronic healthcare records.

**Results:**

Median age was 44.1 years and diabetes duration 20.9 years. The prevalence of CKD stages G1, G2, G3 and G4 and end-stage renal disease (ESRD) was 64.0%, 29.3%, 5.4%, 0.6%, 0.7%, respectively. Micro/macroalbuminuria prevalence was 8.6% and 3.0%, respectively. The incidence rate of ESRD was 2.5 (95% CI 1.9, 3.2) per 1000 person-years. The majority (59%) of those with chronic kidney disease stages G3–G5 did not have albuminuria on the day of recruitment or previously. Over 11.6 years of observation, the median annual decline in eGFR was modest at −1.3 ml min^−1^ [1.73 m]^−2^ year^−1^ (interquartile range [IQR]: −2.2, −0.4). However, 14% experienced a more significant loss of at least 3 ml min^−1^ [1.73 m]^−2^. These decliners had more cardiovascular disease (OR 1.9, *p* = 5 × 10^−5^) and retinopathy (OR 1.3 *p* = 0.02). Adding HbA_1c_, prior cardiovascular disease, recent mean eGFR and prior trajectory of eGFR to a model with age, sex, diabetes duration, current eGFR and ACR maximised the prediction of final eGFR (*r*^2^ increment from 0.698 to 0.745, *p* < 10^−16^). Attempting to model nonlinearity in eGFR decline or to detect latent classes of decliners did not improve prediction.

**Conclusions:**

These data show much lower levels of kidney disease than historical estimates. However, early identification of those destined to experience significant decline in eGFR remains challenging.

**Electronic supplementary material:**

The online version of this article (10.1007/s00125-019-05052-z) contains peer-reviewed but unedited supplementary material, which is available to authorised users.

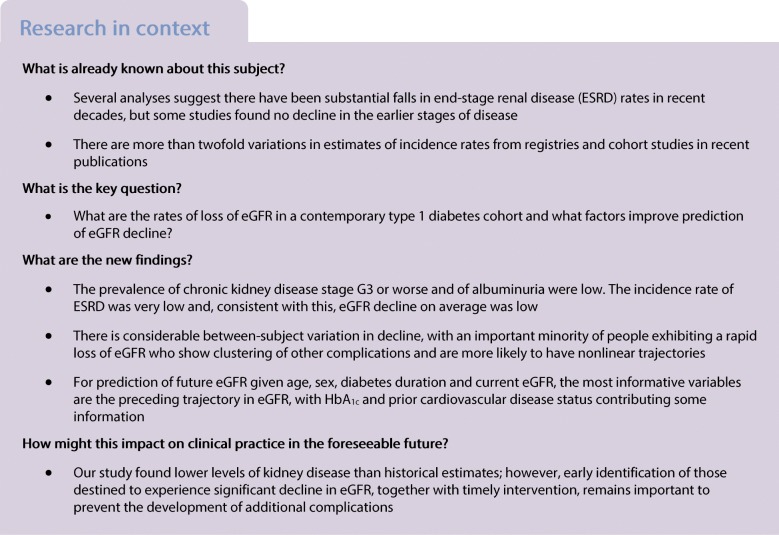



## Introduction

The purpose of this study was to describe the levels and predictors of eGFR decline in a large (*N* = 5777) contemporary population-representative cohort with type 1 diabetes. As well as the ongoing burden of end-stage renal disease (ESRD), earlier stages of chronic kidney disease (CKD) remain one of the strongest predictors of reduced life expectancy in type 1 diabetes [1]. However, there are gaps in our understanding of current risks of CKD both at the population and individual level. Several analyses suggest that there have been substantial falls in ESRD rates in recent decades [2–4] but some cohort studies found no decline in the earlier stages of disease [5]. There are more than twofold variations in estimates of incidence rates of ESRD from registries and cohort studies in recent publications [2, 4, 6–8]. These variations may in part reflect differences in population coverage, sampling criteria and calendar time period covered. Given the substantial advances in diabetes care over the past decades, more data on the current prevalence of renal disease and current rates of decline in eGFR in type 1 diabetes are needed.

In addition to population-level estimates of absolute risk, being able to predict those most at risk of rapid renal function decline at the individual level is also of importance to enable clinicians to optimise diabetes control, cardiovascular risk management and planning for future renal replacement therapy (RRT). Analyses from many years of follow-up of the Joslin cohorts showed that those who progress to ESRD are a subset with steep linear decline in eGFR trajectory [7, 9] whereas other studies report that some degree of renal function decline is almost universal at long durations of type 1 diabetes [5].

Using a recently (2010–2013) established population-representative cohort [10] of one third of all adults with type 1 diabetes in Scotland, here we sought to: (1) describe the contemporary cross-sectional prevalence of CKD stages and albuminuria by duration of diabetes; (2) describe the contemporary rates of decline of eGFR observed over a median of 10 years of observation; and (3) develop and cross-validate a predictive model of prospective decline over a 5-year follow-up period.

## Methods

### Population studied

The Scottish Diabetes Research Network Type 1 Bioresource (SDRNT1BIO) is a recently generated cohort study of 6127 people with type 1 diabetes [10]. In brief, during 2010–2013 we examined 1/3 of all those aged 16 years and over with type 1 diabetes in Scotland, and obtained blood and urine samples. Recruitment was through primary care and the network of hospital diabetes clinics that deliver, as a minimum, annual clinical review for those with type 1 diabetes; care for type 1 diabetes in Scotland remains secondary-care based, with fewer than 10% being managed in primary care only. We also recruited patients from renal clinics to capture those on dialysis who may attend regular diabetes clinics less frequently once ESRD has supervened. At participating clinics we systematically evaluated each patient appointment list for the subsequent week, and as many eligible patients as could be seen on the day were invited to take part. Full details of recruitment and cohort representativeness can be found in the cohort profile [10]. We included those with a clinical label of type 1 diabetes but also verified that they had started insulin within a year of diagnosis and had no prolonged use of oral hypoglycaemic drugs, which would be more consistent with type 2 diabetes. Although it is increasingly recognised that autoimmune diabetes can develop at any age, for consistency with other studies here we restricted the analysis to those with an age of onset below 50 years.

We linked study day (i.e. day of recruitment) data to the extensive retrospective and prospective electronic healthcare record for diabetes, which has been used with national coverage in Scotland since 2004 [1]. This captures all issued prescriptions and clinical recordings of all measures made, such as blood pressure and laboratory tests. We also linked to other routine health-related data including the Scottish Renal Registry, hospital admissions, routine biochemistry laboratory data and death data. This yielded data spanning 2006–2017.

For each participant, the evaluable person-time started when that person first became registered and observable in the national record (which was generally in 2006 for those with prevalent diabetes at that time, or diagnosis date for incident cases of diabetes thereafter). Follow-up was to the last available date from the Scottish Care Information-Diabetes Collaboration (SCI-Diabetes) (18 August 2017) or date of death or date of incident RRT, whichever came first. Thus, we used data both retrospective to the ‘study day’ on which the individual was physically examined (between December 2010 and November 2013) and data prospective to that date.

Missing values were imputed to the median for continuous variables, and to the majority class for categorical variables.

### Serum creatinine, eGFR and albuminuria

Serial serum creatinine data from the routine clinical laboratory biochemistry data in SCI-Diabetes were used to calculate the CKD-Epidemiology Collaboration (CKD-EPI) eGFR values [11]. Values were first normalised to a single standard over time. This used creatinine performance using data from the UK NEQAS external quality assessment scheme (https://birminghamquality.org.uk/eqa-programmes/gfr/), which the contributing National Health Services (NHS) Laboratories had consented to the use of, thus adjusting for changes in laboratory methods and laboratory drifts over time. Values that were coincident with hospital admissions were excluded. In addition, we validated these electronic record-derived creatinine values by showing that those taken near to the study examination day were very strongly correlated (*r* = 0.86) with a directly measured serum creatinine level from a central laboratory using an isotope dilution mass spectrometry (IDMS)-traceable modified Jaffe method on the Roche P800 platform (Roche, Basel, Switzerland). We defined ESRD as being in receipt of RRT or having an eGFR ≤15 ml min^−1^ [1.73 m]^−2^ (G5). We used the KDIGO 2012 [12] definitions of CKD stages according to ranges of eGFR in ml min^−1^ [1.73 m]^−2^: G1: >90; G2: 60–90; G3: 30–60; G4: 15–30; G5: ≤15.

Similarly, longitudinal urinary albumin/creatinine ratio (ACR) was captured from the routine clinical laboratory data. Clinical record data close to the study day were highly correlated (*r* = 0.73) with ACR measured in paired urine samples with the first taken at the study day and the second several days later using the ADVIA 2400 immunoturbidimetric method for albumin and the ADVIA 2400 enzymatic method for creatinine (Siemens, Munich, Germany). At any time point, albuminuric status was defined as normo-, micro- or macroalbuminuric according to ACR falling in the intervals 0–3.39, 3.39–33.9, or >33.9 mg/mmol, based on the most recent available albuminuria measurement, provided there was no contradictory record of that stage in the preceding or subsequent 90 days, such that transient changes in albuminuria readings were ignored. Therefore, someone who transited from normo- to microalbuminuria but then had another normoalbuminuria measurement within 90 days was assigned as having been normoalbuminuric across that period.

### Summarising trajectories of eGFR and assessing their linearity

We first computed a simple person-specific mean of all available eGFR readings collected in the 2 years up to the day the person was recruited into SDRNT1BIO (the study day). Using 5 years of data retrospective to the study day we also estimated a summary trajectory of eGFR for each individual as a linear regression model, the slope of which is the average effect of time on eGFR for that individual (see electronic supplementary material (ESM) Methods). We also computed the overall slope, i.e. not restricting to eGFR recordings retrospective to the study day. Furthermore, we examined whether eGFR decline is indeed linear or not, and tested other approaches to fitting eGFR trajectories such as using linear mixed models with and without stochastic processes and attempting to detect latent classes of decliners (ESM Methods).

### Construction of a predictive model of final eGFR

After adjustment for age, sex, diabetes duration and follow-up time, we examined univariate associations with final eGFR of clinical factors that have been reported in previous clinical models of renal function decline in type 1 and type 2 diabetes [13]. The clinical variables considered were: diastolic BP (DBP), systolic BP (SBP), HbA_1c_, HDL-cholesterol, total cholesterol, BMI, smoking status (ever/never), use of ACE inhibitors (ACEis) or angiotensin receptor blockers (ARBs), prior occurrence of cardiovascular disease (CVD) events, any retinopathy based on annual screening using retinal photography, and diagnosis of diabetes at age 10–16 years, all recorded at study day. Associations were examined with and without adjustment for study day eGFR and study day ACR.

We then compared the performance of a base model containing age, sex, diabetes duration, follow-up time, and study day eGFR and ACR to two forward selection models constructed as follows:

- Model 1: involved selection across clinical covariate data available;
- Model 2: involved selection across the clinical covariates but also the 2 year mean of eGFR, since this may be less noisy and more predictive than a one-off eGFR measure at the study day; a term of the form retrospective slope × study day eGFR, since the way current eGFR relates to future eGFR may depend on how quickly that current eGFR was achieved; terms for retrospective slope × follow-up time, and study day eGFR × follow-up time, since their impact on future eGFR may also alter with longer follow-up time [14].

Analyses were performed in R version 3.6.1. We used the nestfs package (version 1.0: https://CRAN.R-project.org/package=nestfs) to perform a cross-validated forward selection over the different sets of variables. This approach selects variables based on an approximate false discovery rate computed from the sampling distribution of differences in validation log-likelihood between models with and without each additional variable, obtained across 30 inner cross-validation folds. The procedure was set to terminate when the addition of a variable did not produce an improvement in log-likelihood of at least 2. We fitted the variables selected in a ridge regression model using the glmnet package (version 2.0-18: https://CRAN.R-project.org/package=glmnet) to produce a regression equation with shrunk coefficients, thus less subject to overfitting.

Predictive performance was evaluated with tenfold cross-validation, where model coefficients were learnt (and forward selection performed) on 9/10 of data and used to predict the withdrawn portion. Performance was assessed by computing the squared Pearson and Spearman correlations (*r*^2^) between the observed eGFR achieved at the end of follow-up and that predicted by each model, averaged over the cross-validation folds.

## Results

Of the 6127 people recruited in the SDRNT1BIO cohort, we first restricted the study to the 5777 individuals with age of onset below 50 years and an eGFR reading at recruitment. We used this subset to present the prevalence of albuminuria and eGFR stages.

For the rest of the analyses, we excluded 149 participants with less than 6 months of follow-up, and a further 506 who had fewer than three eGFR measures spanning over 2 years in the 5 years immediately preceding their enrolment into the study, as their eGFR trajectories could not be reliably estimated. Therefore, we used a set of 5122 people when fitting models for prediction of eGFR decline.

### Prevalence of albuminuria and eGFR stages

Table 1 shows the participant characteristics at the study day (when individuals were recruited into SDRNT1BIO), overall and stratified by CKD stage. Prevalence of CKD stages is reported in the column headings of Table 1.

**Table 1:** Participant characteristics at study day stratified by CKD stage

Covariate	Missing	G1 (*N* = 3699, 64.0%)	G2 (*N* = 1691, 29.3%)	G3 (*N* = 312, 5.4%)	G4 (*N* = 36, 0.6%)	All (*N* = 5777, 100%)
Main characteristics						
Age (years)	–	38.5 (28.1, 47.5)	52.1 (43.8, 60.4)	61.7 (53.8, 69.0)	55.0 (45.6, 64.3)	44.1 (32.4, 53.9)
Sex (female), %	–	40.7	47.6	52.2	36.1	43.3
Diabetes duration (years)	–	16.9 (9.1, 26.6)	26.8 (17.7, 36.2)	35.7 (27.3, 45.3)	38.1 (27.6, 44.1)	20.9 (11.6, 31.5)
Diabetes onset before 16 years, %	6	43.0	30.1	29.2	41.7	38.5
Observability						
Total observed time (years)	–	10.4 (9.0, 10.9)	10.7 (10.1, 11.1)	10.9 (10.3, 11.3)	11.1 (9.3, 11.3)	10.5 (9.5, 11.1)
Retrospective study length (years)	–	5.3 (4.4, 6.0)	5.5 (5.0, 6.2)	5.6 (5.1, 6.2)	5.5 (4.9, 5.9)	5.4 (4.7, 6.1)
Prospective study length (years)	–	4.9 (4.1, 5.5)	5.1 (4.3, 5.6)	5.1 (4.1, 5.8)	5.2 (4.6, 5.9)	5.0 (4.2, 5.6)
Retrospective creatinine readings (n)	–	9 (6, 13)	11 (7, 16)	15 (10, 24)	28 (13, 40)	10 (6, 14)
Prospective creatinine readings (n)	–	7 (5, 10)	9 (6, 13)	14 (9, 24)	28 (12, 36)	8 (5, 12)
Kidney function						
ACR (mg/mmol)	281	0.4 (0.2, 0.8)	0.4 (0.2, 1.3)	1.1 (0.4, 10.7)	37.7 (3.3, 83.6)	0.4 (0.2, 1.0)
Albuminuric status (normo/micro/macro), %	281	92.1/6.8/1.1	86.8/10.1/3.1	65.3/18.2/16.5	28.6/25.7/45.7	88.4/8.6/3.0
Last ACR at follow-up (mg/mmol)	180	1.0 (0.5, 2.2)	1.0 (0.5, 3.4)	4.1 (1.2, 21.4)	39.6 (8.1, 118.1)	1.0 (0.6, 3.0)
eGFR (ml min^−1^ [1.73 m]^−2^)	–	106.2 (98.8, 115.8)	79.9 (72.0, 85.7)	49.9 (41.7, 55.5)	24.8 (21.5, 27.1)	97.8 (83.0, 109.9)
Mean eGFR over past 2 years (ml min^−1^ [1.73 m]^−2^)	76	108.5 (100.3, 117.3)	86.5 (77.9, 94.7)	56.1 (48.3, 64.5)	28.7 (24.3, 32.8)	101.1 (87.7, 112.4)
Last eGFR at follow-up (ml min^−1^ [1.73 m]^−2^)	–	103.9 (94.8, 112.8)	81.6 (70.2, 91.9)	47.8 (34.6, 59.2)	10.0 (10.0, 19.7)	96.3 (80.8, 107.8)
Overall eGFR slope (ml min^−1^ [1.73 m]^−2^ year^−1^)	58	−1.2 (−2.1, −0.4)	−1.3 (−2.3, −0.4)	−2.0 (−3.5, −1.0)	−2.9 (−5.2, −1.4)	−1.3 (−2.2, −0.4)
Retrospective eGFR slope (ml min^−1^ [1.73 m]^−2^ year^−1^)	562	−1.2 (−3.2, 0.6)	−1.6 (−3.9, 0.4)	−2.7 (−5.0, −1.0)	−3.7 (−6.9, −2.3)	−1.4 (−3.6, 0.5)
eGFR decline band (stable/moderate/fast), %	58	87.9/9.2/2.9	86.5/9.9 8/3.7	67.0/19.9/13.1	52.8/19.4/27.8	85.9/10.0/4.1
Other covariates						
HbA_1c_ (mmol/mol)	24	69 (61, 81)	68 (60, 79)	70 (61, 81)	74 (64, 79)	69 (61, 80)
HbA_1c_ (%)	24	8.5 (7.7, 9.6)	8.4 (7.6, 9.4)	8.6 (7.7, 9.6),	9.0 (8.1, 9.4)	8.5 (7.7, 9.5)
HDL-cholesterol (mmol/l)	332	1.5 (1.2, 1.7)	1.5 (1.3, 1.9)	1.4 (1.2, 1.8)	1.5 (1.1, 1.8)	1.5 (1.2, 1.8)
Total cholesterol (mmol/l)	174	4.6 (4.0, 5.3)	4.5 (3.9, 5.1)	4.3 (3.7, 4.9)	4.3 (3.9, 4.8)	4.5 (4.0, 5.2)
Body mass index (kg/m^2^)	46	26.1 (23.3, 29.3)	27.2 (24.5, 30.4)	27.1 (24.1, 31.1)	30.4 (26.6, 33.4)	26.5 (23.7, 29.7)
DBP (mmHg)	31	76 (69, 82)	75 (68, 81)	70 (62, 78)	71 (63, 81)	75 (68, 82)
SBP (mmHg)	31	127 (118, 137)	132 (121, 143)	137 (122, 147)	143 (129, 161)	129 (119, 140)
Ever smoker, %	1	58.7	65.2	69.6	69.4	61.3
Any retinopathy, %	111	60.0	72.1	83.2	94.3	65.2
Prior CVD, %	–	3.3	10.8	29.8	22.2	7.3
On any anti-hypertensive treatment, %	–	25.8	52.2	86.5	97.2	37.6
On ACEi or ARB, %	–	23.7	47.5	78.2	83.3	34.2

We report frequency (as %) for categorical variables and median (IQR) for continuous variables

CKD stages are defined according to ranges of eGFR in ml min^-1^ [1.73m]^-2^ : G1: > 90; G2: 60–90; G3: 30–60; G4: 15–30

Participants with ESRD (eGFR ≤ 15 [G5] or RRT; *N* = 39, 0.7%) are not reported as a separate column

Only 6.7% of people had CKD stage G3 or worse, including just 35 (0.6%) on RRT and four having an eGFR ≤15 (G5) and not on RRT (so 0.7% for ESRD prevalence altogether).

The prevalence of albuminuria in this population-representative sample was low at 8.6% for microalbuminuria and 3.0% for macroalbuminuria. The majority of participants with CKD stage G3 (65.3%) and almost one third of those at stage G4 (28.6%) did not have albuminuria on the day of recruitment or previously. Among participants with albuminuria the prevalence of CKD stages G3–G5 was 23% (Table 2). Overall 62.4% of participants were not on any anti-hypertensive drugs, including ACEis and ARBs.

ESM Table 1 shows the participant characteristics stratified by diabetes duration bands. As shown, even among those with more than 30 years of diabetes duration the median eGFR was 85 ml min^−1^ [1.73 m]^−2^, with 75% having an eGFR of 69 ml min^−1^ [1.73 m]^−2^ or more. The prevalence of albuminuria was 15.2% in those with more than 30 years duration and 19.1% in those with more than 40 years duration.

### eGFR trajectories

As shown in Table 1 we had a large number of eGFR measurements per person with a median of 18 measurements per person, 10 preceding the study day and 8 from then onwards, enabling us to model trajectories. Table 2 shows the study day characteristics by albuminuric status. Among participants with micro- or macroalbuminuria, 22.6% and 63.8% respectively were decliners, compared with 11.1% of those normoalbuminuric at the study day.

**Table 2:** Participant characteristics at study day stratified by albuminuric status

Covariate	Normoalbuminuric (*N* = 4857, 88.4%)	Microalbuminuric (*N* = 474, 8.6%)	Macroalbuminuric (*N* = 165, 3.0%)
Main characteristics			
Age (years)	44.1 (32.5, 53.7)	46.8 (35.6, 57.9)	47.6 (38.6, 55.1)
Sex (female), %	43.4	39.5	40.6
Diabetes duration (years)	20.4 (11.0, 31.1)	25.9 (17.1, 36.5)	25.9 (20.3, 35.6)
Diabetes onset before 16 years, %	36.4	47.3	49.7
Kidney function			
ACR (mg/mmol)	0.4 (0.2, 0.7)	7.6 (4.5, 14.5)	75.1 (52.1, 106.0)
Last ACR at follow-up (mg/mmol)	1.0 (0.5, 2.0)	8.2 (3.2, 26.1)	46.1 (14.5, 108.0)
eGFR (ml min^−1^ [1.73 m]^−2^)	98.4 (85.2, 110.1)	89.7 (68.1, 105.6)	63.7 (36.3, 88.6)
Mean eGFR over past 2 years (ml min^−1^ [1.73 m]^−2^)	101.7 (89.4, 112.4)	94.4 (74.4, 109.6)	74.3 (47.4, 92.8)
Last eGFR at follow-up (ml min^−1^ [1.73 m]^−2^)	97.3 (83.8, 108.1)	85.7 (64.0, 101.4)	39.8 (10.0, 72.4)
CKD stage (G1/G2/G3/G4/G5), %	66.3/29.4/4.0/0.2/0.1	50.0/35.0/11.4/1.9/1.7	23.0/30.9/29.7/9.7/6.7
Overall eGFR slope (ml min^−1^ [1.73 m]^−2^ year^−1^)	−1.2 (−2.1, −0.4)	−1.6 (−2.8, −0.7)	−3.9 (−7.0, −2.2)
Retrospective eGFR slope (ml min^−1^ [1.73 m]^−2^ year^−1^)	−1.3 (−3.4, 0.5)	−1.6 (−3.7, 0.5)	−4.1 (−7.8, −1.2)
eGFR decline band (stable/moderate/fast), %	88.9/9.0/2.1	77.4/15.5/7.1	36.2/23.8/40.0
Other covariates			
HbA_1c_ (mmol/mol)	68 (60, 79)	76 (65, 90)	80 (70, 98)
HbA_1c_ (%)	8.4 (7.6, 9.4)	9.1 (8.1, 10.4)	9.5 (8.6, 11.1)
HDL-cholesterol (mmol/l)	1.5 (1.2, 1.8)	1.4 (1.1, 1.7)	1.4 (1.1, 1.6)
Total cholesterol (mmol/l)	4.5 (4.0, 5.2)	4.7 (4.0, 5.4)	4.7 (4.1, 5.7)
BMI (kg/m^2^)	26.4 (23.8, 29.6)	27.1 (23.4, 30.5)	26.7 (24.2, 31.9)
DBP (mmHg)	75 (68, 81)	74 (68, 82)	78 (70, 86)
SBP (mmHg)	128 (119, 139)	132 (120, 145)	142 (130, 157)
Ever smoker, %	60.1	69.2	72.1
Any retinopathy, %	62.7	82.3	91.8
Prior CVD, %	6.0	13.9	21.2
On any anti-hypertensive treatment, %	33.7	68.1	81.8
On ACEi or ARB, %	30.8	61.4	74.5

We report frequency (as %) for categorical variables and median (IQR) for continuous variables

This table is limited to the 5496 participants with non-missing albuminuric status at the study day

Albuminuric status is defined according to ranges of ACR in mg/mmol: normoalbuminuric: 0–3.39; microalbuminuric: 3.39–33.9, macroalbuminuric: >33.9

Considering Table 3, the majority of individuals had quite stable or very slowly declining eGFR, but a small minority had moderate or fast decline defined as an eGFR slope between −3 and −5, or <−5, ml min^−1^ [1.73 m]^−2^, respectively. The prevalence of these decliners was highest among those with lower attained CKD stage by study day. However, most decliners still had eGFR above 60 ml min^−1^ [1.73 m]^−2^ (CKD stages G1 and G2) at the study day, i.e. the largest declines are seen in those with high initial eGFRs at baseline. As shown in Table 3, participants with moderate or fast decline were slightly younger, more likely to be female, with younger age of onset and shorter duration of diabetes than individuals with stable eGFR. Although albuminuria was strongly associated with decline, the majority of moderate and fast decliners were normoalbuminuric (79.8% and 51.5%, respectively). Adjusted for age, sex and diabetes duration, they had significantly more CVD (OR = 1.9, *p* = 5 × 10^−5^) and retinopathy (OR = 1.3, *p* = 0.02). Even when restricted to people whose eGFR was above 60 ml min^−1^ [1.73 m]^−2^ (CKD stages G1 and G2) at the study day, a higher prevalence of CVD was apparent (OR = 2.0, *p* = 3 × 10^−4^).

**Table 3:** Participant characteristics at study day stratified by eGFR decline band

Covariate	Stable eGFR (*N* = 4915, 85.9%)	Moderate decline (*N* = 572, 10.0%)	Fast decline (*N* = 232, 4.1%)	*p* value
Main characteristics				
Age (years)	44.7 (33.7, 54.1)	40.7 (26.6, 51.3)	40.5 (26.4, 51.6)	5.1 *×* 10^*−*10^
Sex (female), %	42.2	53.1	43.5	8.6 *×* 10^*−*6^
Diabetes duration (years)	21.2 (11.7, 31.7)	20.0 (10.7, 30.7)	18.8 (11.7, 27.8)	6.9 *×* 10^*−*2^
Diabetes onset before 16 years, %	36.7	47.7	52.6	3.9 *×* 10^*−*8^
Kidney function				
ACR (mg/mmol)	0.4 (0.2, 0.9)	0.6 (0.3, 2.1)	3.6 (0.4, 63.8)	<10^−16^
Albuminuric status (normo/micro/macro), %	91.1/7.7/1.2	79.8/13.2/7.0	51.5/16.5/32.0	<10^−16^
Last ACR at follow-up (mg/mmol)	1.0 (0.5, 2.5)	1.3 (0.6, 4.4)	4.4 (1.0, 69.5)	<10^−16^
eGFR (ml min^−1^ [1.73 m]^−2^)	98.1 (84.6, 109.6)	96.7 (76.4, 113.0)	87.3 (54.6, 108.7)	<10^−16^
Mean eGFR over past 2 years (ml min^−1^ [1.73 m]^−2^)	101.3 (88.7, 111.9)	101.2 (82.6, 114.1)	93.6 (66.8, 113.9)	<10^−16^
Last eGFR at follow-up (ml min^−1^ [1.73 m]^−2^)	97.7 (84.6, 108.3)	85.3 (62.8, 103.8)	54.5 (15.6, 86.9)	*<*10^*−*16^
CKD stage (G1/G2/G3/G4/G5), %	65.6/29.6/4.3/0.4/0.1	58.9/29.0/10.9/1.2/0.0	45.3/26.7/17.7/4.3/6.0	*<*10^*−*16^
Overall eGFR slope (ml min^−1^ [1.73 m]^−2^ year^−1^)	−1.0 (−1.7, −0.3)	−3.6 (−4.2, −3.3)	−6.8 (−8.8, −5.6)	*<*10^*−*16^
Retrospective eGFR slope (ml min^−1^ [1.73 m]^−2^ year^−1^)	−1.1 (−2.9, 0.7)	−4.0 (−6.3, −2.2)	−6.8 (−10.5, −4.4)	*<*10^*−*16^
Other covariates				
HbA_1c_ (mmol/mol)	69 (60, 79)	71 (62, 84)	81 (69, 100)	<10^*−*16^
HbA_1c_ (%)	8.5 (7.6, 9.4)	8.6 (7.8, 9.8)	9.6 (8.5, 11.3)	<10^*−*16^
HDL-cholesterol (mmol/l)	1.5 (1.2, 1.8)	1.4 (1.2, 1.7)	1.4 (1.1, 1.8)	5.6 *×* 10^*−*4^
Total cholesterol (mmol/l)	4.5 (4.0, 5.2)	4.7 (4.1, 5.2)	4.7 (4.0, 5.4)	3.4 *×* 10^*−*2^
BMI (kg/m^2^)	26.6 (23.8, 29.7)	26.4 (23.5, 30.2)	25.1 (22.4, 28.8)	9.2 *×* 10^*−*1^
DBP (mmHg)	76 (69, 82)	75 (68, 81)	75 (67, 84)	7.1 *×* 10^*−*1^
SBP (mmHg)	129 (119, 140)	128 (119, 139)	130 (118, 144)	2.0 × 10^−5^
Ever smoker, %	61.8	57.4	62.9	3.1 *×* 10^*−*1^
Any retinopathy, %	64.9	66.4	69.3	2.0 *×* 10^*−*2^
Prior CVD, %	6.7	7.7	17.2	5.2 *×* 10^*−*5^
On any anti-hypertensive treatment, %	37.1	37.8	49.1	5.3 *×* 10^*−*9^
On ACEi or ARB, %	34.0	33.7	42.2	2.8 *×* 10^*−6*^

We report frequency (as %) for categorical variables and median (IQR) for continuous variables

This table is limited to the 5719 participants with non-missing eGFR decline band at study day

*p* values are for the difference in means or proportions between participants with stable eGFR and those with moderate or fast decline (crude *p* value for main characteristics, *p* value from models adjusted for age, sex and diabetes duration for kidney function and other covariates). For albuminuric status and CKD stage, models compare the first category (normo and G1, respectively) to all others

eGFR decline bands are defined according to ranges of overall slope in ml min-1 [1.73m]^-2^ year^-1^: stable eGFR: *≥ −*3; moderate decline: between *−*3 and *−*5; fast decline: *< −*5

The overall incidence rate of ESRD was 2.5 (95% CI 1.9, 3.2) per 1000 person-years (26,761 person-years, 67 incident cases). At the study day, the incidence rate of ESRD was 0.5 (95% CI 0.2, 0.9) per 1000 person-years in normoalbuminuric people (22,723 person-years), 5.0 (95% CI 2.5, 9.0) in those with microalbuminuria (2180 person-years), and 54.9 (95% CI 38.6, 75.6) in those with macroalbuminuria (674 person-years). The incidence rates of the composite of ESRD or death by any cause were 8.5 (95% CI 7.3, 9.8), 30.3 (95% CI 23.4, 38.5) and 87.5 (95% CI 66.6, 112.9) per 1000 person-years for normo-, micro- and macroalbuminuria, respectively (overall rate was 12.6 [95% CI 11.3, 14.1], 338 incident cases).

### Prediction of eGFR decline

**Fig. 1:**
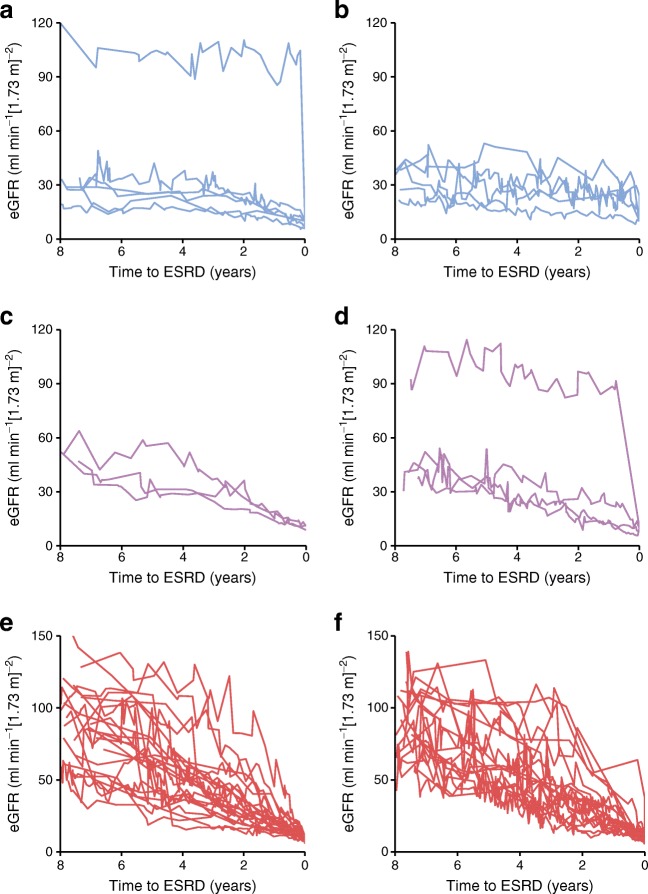
Longitudinal trajectories of eGFR for individuals who reached ESRD during follow-up, stratified by their eGFR decline band and sex. (**a**) Stable eGFR, male, *n*=6. (**b**) Stable eGFR, female, *n*=5. (**c**) Moderate decline, male, *n*=3. (**d**) Moderate decline, female, *n*=4. (**e**) Fast decline, male, *n*=27. (**f**) Fast decline, female, *n*=22. The figure was interrupted at 8 years prior to ESRD, as beyond that only a limited number of participants had eGFR records available

Figure 1 shows the eGFR trajectories observable in those with incident ESRD grouped by eGFR decline band and sex. While many people progress slowly, in some there is a much more precipitous decline. These plots emphasise the challenges of predicting future ESRD in type 1 diabetes and how to identify well in advance those that will experience moderate or fast decline. To this end we attempted a range of modelling approaches, but a simple linear model performed as well as these, and adding nonlinear quadratic terms to such a model did not improve prediction further (ESM Results).

As shown in ESM Table 2, lower final eGFR was associated with older age (β = −0.98) and, after adjustment for age and follow-up time, it was associated with lower study day eGFR (β = 0.76), higher ACR (β = −0.61), being female (β = −4.69) and longer diabetes duration (β = −0.23), all at *p* < 10^−16^. Being diagnosed during teenage years was associated with significantly lower eGFR at follow-up, after adjustment for age, sex and length of follow-up (β = −4.57) (*p* = 1.8 × 10^−15^). When further adjusted for study day eGFR and ACR, only seven of the clinical variables considered were independently associated with final eGFR.

Using forward selection to maximise prediction of final eGFR across clinical variables, only HbA_1c_, being on ACEi or ARB medication, prior CVD, SBP, DBP and HDL-cholesterol were retained in addition to the base model (Table 4). The increment in prediction performance achieved beyond the base model was trivial, with *r*^2^ going from 0.698 to 0.702. However, if summary measures of historical eGFR were considered as in Model 2 of Table 4, there was incremental prediction information especially in the 2-year mean of eGFR, while the only clinical covariates retained were HbA_1c_ and prior CVD. The increment in prediction with this model was greater, with *r*^2^ going from 0.698 to 0.745. The ridge regression equation for Model 2 is shown in the footnote to Table 4.

**Table 4:** Cross-validated performance of baseline and forward selection models and variables selected for prediction of achieved eGFR

Selection order	Variable selected	Difference in log-likelihood	Coefficient	*p* value
Baseline model (Pearson *r*^2^: 0.698; Spearman *r*^2^: 0.670)				
Age, sex, diabetes duration, eGFR, ACR, follow-up time		–	–	–
Forward selection Model 1 (Pearson *r*^2^: 0.702; Spearman *r*^2^: 0.670)				
1	HbA_1c_ (mmol/mol)	31.6	−0.1	1.3 × 10^−15^
2	On ACEi or ARB	7.3	−1.3	2.1 × 10^−03^
3	Prior CVD	4.3	−2.0	4.9 × 10^−03^
4	SBP (mmHg)	3.0	−0.1	4.6 × 10^−05^
5	DBP (mmHg)	3.1	0.1	4.4 × 10^−03^
6	HDL-cholesterol (mmol/l)	3.7	1.4	9.1 × 10^−04^
Forward selection Model 2 (Pearson *r*^2^: 0.745; Spearman *r*^2^: 0.717)				
1	Mean eGFR over past 2 years (ml min^−1^ [1.73 m]^−2^ year^−1^)	387.1	0.5	<10^−16^
2	HbA_1c_ (mmol/mol)	33.0	−0.1	3.9 × 10^−16^
3	Prior CVD	4.4	−2.1	9.4 × 10^−04^
4	Retrospective slope *×* follow-up time / 100	7.4	−4.1	1.7 × 10^−05^

Cross-validated performance of models is reported as the mean squared correlation (Pearson *r*^2^) and mean squared rank correlation (Spearman *r*^2^) between predicted and observed achieved eGFR over 10 cross-validation folds

Variables in forward selection models are listed according to the order of selection

Differences in log-likelihood are reported with respect to the baseline model for the first variable in each model and then incrementally

Regression coefficients and *p* values are reported for the model fitted with all selected variables after adjusting for the variables in the baseline model using ordinary least squares on all data

Ridge regression equation for forward selection Model 2:

46 *−* (0.21 *×* age) *−* (1.2 *×* female sex) *−* (0.087 *×* duration) + (0.3 *×* eGFR) *−* (0.27 *×* ACR) *−* (0.11 *×* follow-up time in years) + (0.36 *×* mean eGFR past 2 years) *−* (0.065 *×* HbA_1c_) *−* (3 *×* prior CVD) *−* (0.0082 *×* retrospective slope *×* follow-up time in years)

## Discussion

In this large contemporary population-representative cohort of people with type 1 diabetes, key findings are that the prevalence of CKD stage G3 or worse and of albuminuria were low even in those with long-standing type 1 diabetes, and that the incidence rate of ESRD and average eGFR decline were low. However, there is considerable between-subject variation in this decline, and we show that there remains an important minority of participants who exhibit a rapid loss of eGFR. Such individuals show clustering of other complications even when eGFR is not yet low. Importantly, while albuminuria is predictive of decline, the majority of moderate or fast decliners are normoalbuminuric. Identifying such individuals while the eGFR has not yet declined to more advanced CKD stages remains a challenge. A model-derived risk equation helps to identify renal decline, with most of the improvement in prediction deriving from using recent mean eGFR rather than relying on a single current eGFR reading.

The prevalence of advanced CKD, any albuminuria and ESRD, and the incidence rate of ESRD we report, are low in comparison with historical estimates but consistent with observed large reductions in albuminuria and ESRD in type 1 diabetes in Europe in recent decades [2, 4, 6, 15, 16]. Our albuminuria prevalence at 11.6% is very similar to the current prevalence of 12% in clinical attendees with type 1 diabetes in the 2017 Swedish National Diabetes Registry annual report (https://www.ndr.nu/#/arsrapportinsert). In the USA, prevalence of albuminuria in the T1D Exchange registry in recent analyses was also low [17, 18], although direct comparisons are hampered by differing recruitment strategies. We found a low incidence rate of ESRD at 2.5 (95% CI 1.9, 3.2) per 1000 person-years. These data are similar to rates of 2–5 per 1000 person-years for those aged 19 years and upwards reported in the Swedish registry study [2] and rate of 2.5 per 1000 person-years in Finland [4]. In Norway and Japan, even lower rates of 0.7–0.8 per 1000 person-years have been reported [3, 6]. Much higher rates of albuminuria and ESRD were reported for long term follow-up of some [5, 19] although not all [20] US cohorts. Large international variations in ESRD rates among people with type 1 diabetes with macroalbuminuria and CKD1–3 at entry were also recently noted [8]. The extent to which these international differences reflect differing selection criteria or calendar time periods or real differences in risk or clinical management remains unclear.

There are few recent studies of progression rates of eGFR in a general cohort of people with type 1 diabetes, not selected on the basis of albuminuria or eGFR. As such we provide useful data on the distribution of eGFR slopes across the population with type 1 diabetes, and which reflect recent management practices. Where comparison is possible, our data have similarities to those from the Joslin cohorts in a number of important respects [9, 21–23]. In those studies, among people with eGFR >60 ml min^−1^ [1.73 m]^−2^ at baseline 9%/22%/51% of those with normo-/micro-/macroalbuminuria had a slope ≤−3 ml min^−1^ [1.73 m]^−2^, very similar to the 10.7%/20.7%/54.5% shown here. Second, despite the importance of albuminuria as a predictor of decline, it remains insensitive in that normoalbuminuria is frequent even among those with CKD stage G3 or worse. This confirms the findings in the Joslin cohorts [22] and reinforces the importance of monitoring eGFR itself, and not just albuminuria, in people with diabetes.

Regarding prediction of future eGFR and the identification of moderate or fast decliners, we first examined whether or not eGFR trajectories are linear across all people. This question is of importance in several ways; if trajectories are linear, then past trajectory can simply be plotted to estimate future eGFR and there is little need for other information. If trajectories are nonlinear for many, then can this be used to identify those declining most rapidly? Although we found that nonlinearity is common at 26.2% of moderate or fast decliners vs 15.7% of the remainder (ESM Results), this difference was not sufficient for classifying decline status nor was modelling the nonlinearity useful in predicting final eGFR. Some of this nonlinearity could reflect periods of acute injury followed by more rapid decline. In the Joslin cohorts an assessment of linearity was made, but retrospectively, among those who had reached ESRD [9]. Nonetheless, similar conclusions are reached—although 40% of the Joslin cohort had trajectories that were nonlinear, this also had an inconsequential impact on predicted time of onset of ESRD.

The most predictive model was a linear one that included age, sex, diabetes duration, current eGFR and ACR, HbA_1c_, prior CVD status and the 2-year mean of eGFR, with prior slope of eGFR adding a little further information. In particular, using mean eGFR over 2 years gave a substantial improvement in predicting future eGFR because single eGFR readings are quite noisy and variable. To translate this prediction model into usefulness in clinics would require that clinical e-health systems compute the predicted eGFR after a given follow-up time, incorporating variables such as prior CVD and HbA_1c_. As the prediction improvement may be too low to incur the costs of formally implementing such a model, a more pragmatic implication is simply that summary views of the mean of recent eGFRs and 5-year slopes should be presented to the clinician alongside current eGFR. Our data further support the use of measures of prior rate of change in eGFR and not just a single baseline eGFR reading when selecting people for entry into randomised trials, such as was recently done in the Preventing Early Renal Loss in Diabetes (PERL) trial [24]. However, prediction of future eGFR remains far from perfect even with the best model, justifying ongoing attempts to identify predictive biomarker panels [25].

Strengths of our study include its contemporaneous nature and its size. An important aspect is the population-representativeness of this cohort of all adults with type 1 diabetes nationally. The prevalence of RRT using the Scottish Renal Registry at recruitment was 0.6%, very similar to the prevalence of 0.82% we calculate in the data available to us on the national population of people with type 1 diabetes using the same entry criteria (age at onset of diabetes <50 years) for the same entry years (H. M. Colhoun, unpublished data). We previously reported a slightly lower prevalence of albuminuria in the SDRNT1BIO cohort than the overall type 1 population nationally [10]. This did not take account of the large percentage of missingness of ACR in the national data (up to 40% in any year), which elevates the apparent prevalence of albuminuria by a few percentage points as missingness is higher in those who are in fact normoalbuminuric. Nonetheless, it is possible that our cohort may have a slightly lower risk profile than the population with type 1 nationally, and as such the rates of incidence of ESRD and eGFR decline may be a slight underestimate.

Our study has limitations, the most important being that our follow-up time is limited. However, in SDRNT1BIO we have routine capture of all clinical data prospectively so that years of follow-up are accruing, allowing further analyses in future. A second limitation is that, apart from the study day, we are using real-world clinical eGFR records, which inevitably will reflect noise due to inter-laboratory variability. For this reason, we accessed the national laboratory quality control data scheme (UK NEQAS) for normalisation, which sends a monthly standards report across the range of eGFR to all laboratories, allowing inter-laboratory variation and drift through time to be quantified and adjusted for.

In conclusion we have shown that the majority of people with type 1 diabetes do not have moderate or fast declining renal disease and that current rates of progression appear low in comparison with historical estimates. This is good news for people with the condition. However, although progressive loss of renal function is less common than in the past, it remains very important. In this regard we note that decliners constitute a group already at elevated risk for CVD and retinopathy even at young ages and while their eGFR still remains >60 ml min^−1^ [1.73 m]^−2^, emphasising the importance of their early identification. Such decliners need vigilant intervention for renal protection and general CVD risk factor control. Their identification could be improved using a formal risk prediction model or at least encouraging clinicians to consider means of recent eGFR measurements and prior slopes alongside the most current eGFR.

## Electronic supplementary material

ESM (PDF 292 kb)

## Data Availability

We do not have governance permissions to share individual level data on which these analyses were conducted since they derive from clinical record data. However, for any bona fide requests to audit the validity of the analyses, the verifiable research pipeline which we operate means that one can request to view the analyses being run and the resulting tabulations by contacting the corresponding author. We are also happy to share summary statistics for those wishing to conduct meta-analyses with other studies.

## Abbreviations

ACEi: ACE inhibitor
ACR: Albumin/creatinine ratio
ARB: Angiotensin receptor blocker
DBP: Diastolic BP
CKD: Chronic kidney disease
CVD: Cardiovascular disease
ESRD: End-stage renal disease
IQR: Interquartile range
RRT: Renal replacement therapy
SBP: Systolic BP
SDRNT1BIO: Scottish Diabetes Research Network Type 1 Bioresource
