# Supplementary material for: Predicting renal disease progression in a large contemporary cohort with type 1 diabetes mellitus
Source: Diabetologia. 2019 Dec 5;63(3):636–47. doi: 10.1007/s00125-019-05052-z (PMC6997248; doi:10.1007/s00125-019-05052-z)
Supplement: Supplementary file 1 — (PDF 292 kb) [file 125_2019_5052_MOESM1_ESM.pdf]

Electronic Supplementary Materials

Predicting Renal Disease Progression in a Large Contemporary Cohort with Type 1 Diabetes Mellitus

ESM Methods

Summarising trajectories of eGFR and assessing their linearity

Serial serum creatinine data from the routine clinical laboratory biochemistry data in SCI-Diabetes was used to calculate the CKD-EPI eGFR [1] using the formula

$$\text{eGFR} = 141 \times \min(\text{Scr}/\kappa, 1)^{\alpha} \times \max(\text{Scr}/\kappa, 1)^{-1.209} \times 0.993^{\text{Age}}[\times 1.018 \text{ if female}][\times 1.159 \text{ if black}],$$

where Scr is serum creatinine in µmol/l, age is expressed in years, κ is 0.7 for females and 0.9 for males, and α is -0.329 for females and -0.411 for males.

Using 5 years data retrospective to study day (the day when participants were recruited into SDRNT1BIO), we estimated a summary trajectory of eGFR separately for each individual. To accomplish that, within each month we first considered the median eGFR readings non-concurrent with hospital admissions. To these data we applied an exponential smoothing with a time window of 1 year according to:

$$s_i = (\text{eGFR}_i + w_i s_{i-1})/(1 + w_i), \quad i = 2, \ldots, n,$$

where s_i is the i-th smoothed eGFR reading for that individual, and $w_i = \max(1 - d_i/365, 0)$ is the weight assigned to the previous terms, which depends on the number of days d_i between two successive eGFR readings, eGFR_{i-1} and eGFR_i.

We then fitted a linear regression model for each participant over the smoothed data, as long as the individual had at least 3 readings over a period of at least 2 years. Therefore, for each person with n smoothed eGFR observations recorded at time t_i (expressed in years from the first reading used), we fitted the following model:

$$s_i = \alpha + \beta t_i + \varepsilon_i, \quad i = 1, \ldots, n,$$

where α and β are intercept and slope, respectively, and $\varepsilon_i \sim N(0, \sigma^2)$ is the random error. Thus, the slope term β is the average annualized effect of time on eGFR for that individual.

To assess the frequency of trajectories that are non-linear, we fitted for each person a model containing a quadratic term for time, of the form:

$$s_i = \alpha + \beta t_i + \gamma t_i^2 + \varepsilon_i, \quad i = 1, \ldots, n.$$

To examine across all individuals the extent to which taking into consideration any departure from linearity is useful for prediction of final eGFR, we compared the adjusted r^2 with respect to the baseline model for a linear regression model of final eGFR with and without a quadratic term across all individuals.

We compared the above approach of computing slopes to using a linear mixed model (LMM) and conditional two-step LMM [2], as well as a linear mixed effects model with non-stationary stochastic processes [3], as implemented in the `lmenssp` R package (version 1.2: https://CRAN.R-project.org/package=lmenssp).

We further formulated the problem as a latent class mixed model, which allows to assign group membership to each participant according to their profile of trajectory. We attempted to fit models with 2 and 3 latent classes using the R package `lcmm` [4] (version 1.7.8: https://CRAN.R-project.org/package=lcmm).

ESM Results

To test whether eGFR decline can be treated as being linear we fitted individual models to test whether quadratic terms improve the fit of eGFR trajectories beyond simple linear terms. In 18.9% there was some improvement in fit of the model of trajectories as evidenced by the quadratic term being statistically significant at $p < 0.01$ (26.2% of participants with an average loss in eGFR of at least 3 ml $\text{min}^{-1}[1.73\text{m}]^{-2}$ year^{-1}, and 15.7% of the remainder). However, when we evaluated how useful including a quadratic term is across all individuals for predicting final eGFR, the improvement in prediction was trivial (r^2 increased from 0.698 to 0.701 only). This was the case both in those with study day eGFR above or below 60 ml $\text{min}^{-1}[1.73\text{m}]^{-2}$, thus the remaining analyses did not include quadratic effects of time on eGFR.

Neither using linear mixed models with or without stochastic processes improved prediction performance. Using a latent class approach to establish the existence of a group with moderate or fast decline, no classes were discoverable.

ESM References

[1] Levey AS, Stevens LA, Schmid CH, Zhang Y, Castro I Alejandro F., Feldman HI, et al. A new equation to estimate glomerular filtration rate. Annals of Internal Medicine 2009;150:604–12.

[2] Sikorska K, Rivadeneira F, Groenen PJ, Hofman A, Uitterlinden AG, Eilers PH, et al. Fast linear mixed model computations for genome-wide association studies with longitudinal data. Statistics in Medicine 2012;32:165–80.

[3] Diggle PJ, Sousa I, Asar Ö. Real-time monitoring of progression towards renal failure in primary care patients. Biostatistics 2015;16:522–36.

[4] Proust-Lima C, Philipps V, Liquet B. Estimation of extended mixed models using latent classes and latent processes: The R package lcmm. Journal of Statistical Software 2017;78:1–56.

ESM Table 1: Participant characteristics at study day stratified by duration bands in years

Covariate	0–15 (N = 1976, 34.2%)	15–30 (N = 2168, 37.5%)	> 30 (N = 1633, 28.3%)	> 40 (N = 627, 10.9%)
**Main characteristics**				
Age (years)	33.7 (24.9, 44.1)	42.5 (33.1, 52.0)	54.7 (47.4, 62.7)	60.0 (53.9, 66.8)
Sex (female), %	40.8	44.0	45.4	46.3
Diabetes duration (years)	8.5 (4.3, 11.7)	21.9 (18.7, 25.8)	37.4 (33.1, 43.4)	45.2 (42.3, 50.0)
Diabetes onset before 16 years, %	22.4	40.5	55.5	64.3
**Kidney function**				
ACR (mg/mmol)	0.4 (0.2, 0.7)	0.4 (0.2, 1.1)	0.5 (0.3, 1.5)	0.6 (0.3, 2.4)
Albuminuric status (normo/micro/macro), %	94.0/5.1/0.9	86.0/9.8/4.2	84.8/11.3/3.9	80.9/14.3/4.8
Last ACR at follow-up (mg/mmol)	1.0 (0.5, 1.8)	1.0 (0.6, 3.3)	1.4 (0.7, 3.9)	1.7 (0.8, 5.6)
eGFR (ml $min^{-1}[1.73m]^{-2}$)	107.3 (95.5, 118.9)	97.2 (83.5, 109.1)	85.5 (69.1, 97.8)	78.3 (60.9, 92.4)
Mean eGFR over past 2 years (ml $min^{-1}[1.73m]^{-2}$)	110.4 (100.2, 120.5)	100.7 (88.5, 111.6)	89.1 (75.0, 100.8)	82.6 (65.6, 94.6)
Last eGFR at follow-up (ml $min^{-1}[1.73m]^{-2}$)	105.4 (94.7, 115.7)	96.2 (81.8, 107.1)	83.2 (66.6, 95.8)	75.4 (57.0, 90.3)
CKD stage (G1/G2/G3/G4/G5), %	83.6/15.8/0.6/0.0/0.1	64.1/30.4/4.3/0.5/0.7	40.3/44.1/12.7/1.5/1.3	30.0/45.8/19.8/2.6/1.9
Overall eGFR slope (ml $min^{-1}[1.73m]^{-2} year^{-1}$)	-1.3 (-2.3, -0.3)	-1.3 (-2.2, -0.5)	-1.2 (-2.1, -0.4)	-1.2 (-2.2, -0.3)
Retrospective eGFR slope (ml $min^{-1}[1.73m]^{-2} year^{-1}$)	-1.4 (-3.7, 0.7)	-1.5 (-3.7, 0.3)	-1.3 (-3.2, 0.5)	-1.4 (-3.2, 0.4)
eGFR decline band (stable/moderate/fast), %	85.1/10.7/4.2	85.4/9.9/4.7	87.7/9.3/3.0	86.7/9.4/3.9
**Other covariates**				
HbA_{1c} (mmol/mol)	70 (61, 84)	70 (61, 81)	68 (60, 77)	67 (60, 76)
HbA_{1c} (%)	8.6 (7.7, 9.8)	8.6 (7.7, 9.6)	8.4 (7.6, 9.2)	8.3 (7.6, 9.1)
HDL-cholesterol (mmol/l)	1.4 (1.2, 1.7)	1.5 (1.2, 1.8)	1.6 (1.3, 1.9)	1.6 (1.3, 1.9)
Total cholesterol (mmol/l)	4.6 (4.0, 5.3)	4.6 (4.0, 5.2)	4.4 (3.8, 5.1)	4.4 (3.8, 5.0)
Body mass index (kg/m^2)	25.7 (22.9, 28.9)	26.7 (24.0, 30.1)	27.1 (24.4, 30.2)	26.9 (24.3, 30.1)
DBP (mmHg)	76 (70, 82)	77 (70, 82)	72 (65, 80)	70 (63, 77)
SBP (mmHg)	125 (117, 135)	129 (119, 139)	134 (124, 145)	136 (125, 149)
Ever smoker, %	55.3	62.6	66.8	67.0
Any retinopathy, %	32.8	81.3	82.8	80.8
Prior CVD, %	1.7	5.1	16.8	25.2
On any anti-hypertensive treatment, %	16.1	39.9	60.6	68.6
On ACEi or ARB, %	14.3	36.4	55.4	61.9

We report frequency (as %) for categorical variables and median (IQR) for continuous variables

ESM Table 2: Univariate associations of relevant clinical variables with achieved eGFR in linear regression models adjusted for the sets of covariates indicated

Covariate	β (95% CI)	p value
**Adjusted for follow-up time**		
Age (years)	-0.98 (-1.01, -0.94)	$< 10^{-16}$
**Adjusted for age and follow-up time**		
eGFR (ml $min^{-1}[1.73m]^{-2}$)	0.76 (0.74, 0.78)	$< 10^{-16}$
ACR (mg/mmol)	-0.61 (-0.65, -0.58)	$< 10^{-16}$
Diabetes duration (years)	-0.23 (-0.28, -0.19)	$< 10^{-16}$
Sex (female)	-4.69 (-5.69, -3.68)	$< 10^{-16}$
**Adjusted for age, sex and follow-up time**		
Diabetes onset before 16 years	-4.57 (-5.70, -3.45)	1.8×10^{-15}
**Adjusted for age, sex, duration, eGFR, ACR and follow-up time**		
HbA_{1c} (mmol/mol)	-0.09 (-0.11, -0.07)	6.8×10^{-16}
Prior CVD	-2.98 (-4.35, -1.60)	2.2×10^{-5}
On ACEi or ARB	-1.71 (-2.52, -0.89)	4.1×10^{-5}
HDL-cholesterol (mmol/l)	1.68 (0.86, 2.50)	6.2×10^{-5}
SBP (mmHg)	-0.03 (-0.06, -0.01)	3.2×10^{-3}
Body mass index (kg/m^2)	-0.09 (-0.16, -0.02)	1.4×10^{-2}
Any retinopathy	-0.82 (-1.61, -0.02)	4.4×10^{-2}
Total cholesterol (mmol/l)	-0.14 (-0.48, 0.20)	4.1×10^{-1}
DBP (mmHg)	0.01 (-0.02, 0.04)	5.6×10^{-1}
Ever smoker	-0.02 (-0.74, 0.70)	9.5×10^{-1}

Scottish Renal Registry Steering Group Members November 2019

Name	Designation	Unit
Dr Samira Bell	Consultant Nephrologist/Chair	Ninewells Hospital
Dr Jamie Traynor	Consultant Nephrologist/ Technical Director	Queen Elizabeth University Hopsital
Dr Wendy Metcalfe	Consultant Nephroligist	Royal Infirmary of Edinburgh
Dr Bruce MacKinnon	Consultant Nephrologist	Queen Elizabeth University Hopsital
Dr David Hughes	Consultant Nephrologist	Royal Hospital for Children
Dr David Walbaum	Consultant Nephrologist	Aberdeen Royal Infirmary
Dr Elaine Spalding	Consultant Nephrologist	Crosshouse Hospital
Mr Ian Currie	Consultant Tx Surgeon	Royal Infirmary of Edinburgh
Mr Marc Clancy	Consultant Tx Surgeon	Queen Elizabeth University Hopsital
Dr Mark Findlay	Consultant Nephrologist	Queen Elizabeth University Hopsital
Dr Michaela Petrie	Consultant Nephrologist	Royal Infirmary of Edinburgh
Dr Peter Thomson	Consultant Nephrologist	Queen Elizabeth University Hopsital
Mrs Rachel Cox	Renal Practice Educator	Crosshouse Hospital
Dr Robert Peel	Consultant Nephrologist	Raigmore Hosital
Dr Vinod Sanu	Consultant Nephrologist	Ninewells Hospital
Dr Shona Methven	Consultant Nephrologist	Aberdeen Royal Infirmary
Dr Sue Robertson	Consultant Nephrologist	Dumfries and Galloway Royal Infirmary
Dr Ilona Shilliday	Consultant Nephrologist	University Hospital Monklands
Dr Zoe Cousland	Consultant Nephrologist	University Hospital Monklands
Ms Angie Doherty	Dietician	Glasgow Royal Infirmary
Dr Arthur Doyle	Consultant Nephrologist	Victoria Hospital
Mrs Jackie McDonald	Data Manager	NHS National Services Scotland
Mrs Jacqueline Campbell	Senior Analyst	NHS National Services Scotland
Prof Corri Black	Director of the Aberdeen Centre for Health Data Sciences	University of Aberdeen
